# Thinking through the micro-politics of disclosure/exposure while researching race and gender: a multi-voice essay on how to manage vulnerability

**DOI:** 10.3389/fsoc.2026.1743053

**Published:** 2026-07-06

**Authors:** Anaïs Ménard, Johanna M. Lukate, Madeline Bass, Karen Amaka Okigbo, Serena Scarabello, Vinícius Venancio, Kezia Aryeetey, Samantha Strohmenger

**Affiliations:** 1 Department of Social and Cultural Anthropology, KU Leuven, Leuven, Belgium; 2 Max Planck Institute for the Study of Religious and Ethnic Diversity, Göttingen, Germany; 3 Research Group “Migration, Identity and Blackness in Europe”, Max Planck Institute for Human Development, Berlin, Germany; 4 Department of Sociology, University of Massachusetts-Boston, Boston, MA, United States; 5 Department of Political and Social Sciences, University of Pavia, Pavia, Italy; 6 Faculty of Social Sciences, Federal University of Goiás, Goiânia, Brazil; 7 Otto-Hahn-Research Group “Gender, Migration and Social Mobility”, Max Planck Institute for Social Anthropology, Halle, Germany; 8 Faculty of Humanities, Department of Social and Cultural Anthropology, Eberhard Karls Universität Tübingen, Tübingen, Germany

**Keywords:** disclosure, exposure, gender, positionality statements, research process, vulnerability

## Abstract

This multi-voice essay emerges from a collective, multidisciplinary workshop on *Researching Race and Gender*, where participants reflected on the micro-politics of disclosure and exposure while researching and writing about race, racism and gender. Drawing on diverse disciplinary backgrounds and field locations, our collaborative reflections explore how researchers navigate the vulnerabilities that come with the demand for transparency in contemporary academic practice. We argue that while a neoliberal research culture invites openness and reflexivity, it often fails to acknowledge the embodied, emotional, and political vulnerabilities that certain acts of disclosure entail. Through an assemblage of personal experiences and critical reflections, we thus ask: *how do researchers deal with the vulnerabilities resulting from processes of disclosure/exposure? And in particular, how do they engage questions of disclosure and exposure in researching race and racism?* In this essay, we then build upon our own experiences around disclosure and exposure, to consider how power relations shape what can be said, revealed, or withheld, and by whom, while offering concrete suggestions and strategies for how to navigate the pressures of disclosure at various stages of the research process.

## 1 Introduction

When doing fieldwork, researchers conducting critical, qualitative research on race and gender often share details of their personal life with their interlocutors. They may be asked, or feel compelled to do so, in some of the conversations they initiate. They may choose to disclose personal information, more generally, to establish rapport based on mutual understanding, reciprocity and trust with the people they work with. These choices of transparency influence the perceptions that interlocutors have of the researcher and the interactions they create with each other. Additionally, questions of identity and positionality are discussed informally, in conversations with colleagues, in supervision meetings, and among researchers who share similar or diverging experiences of navigating race and gender in the field. Over the past three decades, however, there has been a growing push across research disciplines for greater transparency with regards to one’s identity and positionality in academic writing, which is reflected in the growing expectation for positionality and reflexivity statements to be included in scholarly publications. This push is best understood as part of the ‘reflexive turn’ in social science research and efforts to make visible the assumptions and positionalities that shape the production of knowledge. While few researchers would dispute the need to explain their position in the field, the criteria for writing these sections remain unclear. Thus, authors are left to make their own editorial choices, often obscuring how those choices were made behind academic language that smooths over the messy aspects of fieldwork. Nevertheless, amongst themselves, they will share ‘the uncomfortable stuff’: uneasy situations that they encountered in the field, relational difficulties with interlocutors, the social mistakes that they made, the violence that they/their interlocutors experienced, and, critically, the fragmented, context-dependent way that their identity works in the field, with different aspects of their identity and positionality becoming relevant across encounters yet rarely all at once.

This was exactly the situation we found ourselves in as we organized an inter-research group workshop on ‘Researching Gender & Race’ in June 2024 at the Max Planck Institute for the Study of Religious and Ethnic Diversity.^1^ The room welcomed eight researchers with different cultural backgrounds and linguistic skills, coming from various institutions and working in different academic disciplines within the social sciences—anthropology, sociology, Black and Indigenous Studies and social psychology. We also had varied and multi-sited fieldwork experiences around researching topics at the intersection of race and gender, which involved different types of gendered and ethnic/racial positionings in the field, and yet shared common concerns about positionality and reflexivity in the research process.

In this article, we collectively engage these issues by examining the practical implications of our shared and divergent positionalities in order to bring forward reflections on the researchers’ vulnerability and exposure. Our collective is made up of researchers with Black and white heritage (including individuals who identify with both groups), women, and one man. We did not disclose our sexualities or gendered identities beyond pronoun usage. Our ethnicities and nationalities cross the Atlantic, including Africa, Europe, South and North America. These positionalities matter to our research and the workshop discussion, even as their meanings fluctuate across contexts and audiences. As we explore at length below, our shared focus on race and gender is impacted by our own racialized, gendered and sexualized identities, and the different ways they surfaced in our research.

Our collective arose from specific institutional conditions: at the time of the workshop, we were all based, either as formal staff members or guests, at research institutions of the Global North. There were marked differences in our career stages and the stability of our professional situations, which influenced our respective contributions. However, we shared an ‘epistemic location’ (Grosfoguel, 2011) that gave prominence (but not exclusivity) to a common language (i.e., English) and a literary corpus produced in/for the Western world. Consequently, we are aware that this article may reproduce the ‘coloniality of power’ (Quijano, 2000) that it seeks to deconstruct (albeit partially) by decentering perspectives on positionality. Yet it is precisely from this position of ‘institutional centrality’ (Collyer et al., 2019, p. 150) that we wish to articulate central concerns about the vulnerability of researchers who stand at the margins of the invisible norm. Reflecting on the unspoken rules of positionality allows us to further question the methodological asymmetries at the heart of knowledge production.

At the workshop, our main discussion focused on the challenging balance between the researcher’s exposure (in the field and in their writing) and the need to protect personal information through disclosure choices. From an academic perspective, Pillow (2010, p. 270) points out, researchers in the social sciences ‘run the risk of not being confessional enough, to being too confessional and thus not rigorous enough.’ Moreover, the display of scientific rigor and credibility follows specific standards of writing, particularly in peer-reviewed journals, that do not necessarily allow for lengthy and nuanced explanations on positionality. Thus, positionality statements inevitably represent a condensed, edited, stylized—and therefore imperfect—translation of the author’s subject position at that time. How did we, as individual researchers, try to achieve balance between disclosure, exposure, and concealment? How did this relate to issues of academic legitimacy? How did we navigate and manage our personal vulnerabilities in processes of disclosure/exposure?

Positionality is a process that takes its full social meaning in relation to what we have termed the micro-politics of disclosure/exposure: what you reveal (or hide) in certain situations, in writing, and how these micro-politics relate to the researcher’s sense of vulnerability and legitimacy (in the research process and in writing). These issues are particularly relevant to our group because, as individual researchers, we all practice long-term ethnography as our primary or secondary research method. As Lumsden (2019, p. 18) shows, the reflexive turn in the social sciences emerged as a response to ‘a double crisis of representation and legitimation’ that questioned both the reliability of the ethnographic practice and the production of scientifically valid texts. Reflexivity appeared as an avenue to expose the conditions of research and ‘explore the biases and incentives to which [researchers] are otherwise oblivious’ (Bukamal, 2022, p. 329). Within this reflexive practice, the researcher’s awareness of their subject position became a major tool for data collection and analysis. Nevertheless, while positionality now occupies a central place in ethnographic and qualitative research practice, the fluid, dynamic and context-dependent nature of researcher identities can never be fully captured, either in interactions or in writing (Thomson and Gunter, 2010).

From this perspective, we argue that the research process, at its various stages, involves *choices of transparency* that depend on the context of the research and the researcher’s preferences, sensibilities and strategies. Yet these choices and the social and psychological mechanisms feeding them are often ignored. Therefore, our purpose is not to discuss positionality as such, but to offer the reader (and in particular early career researchers) strategies for managing exposure and disclosure as we have employed them ourselves while researching and writing on race and gender. In doing so, we would like to highlight the concept of disclosure as ‘exposure.’

In this essay, we present the product of a collective multidisciplinary reflexive process, as we thought through the micro-politics of disclosure/exposure while researching race and gender, from fieldwork to ethnographic writing. We also want to give examples of how we can struggle, differently, to present aspects of our identity, by comparing positionality statements that we have written at different periods, in different articles. These examples will illustrate the difficulty in achieving the balance between offering too much detail or not enough, and framing identities (of oneself, one’s family, one’s heritage) in acceptable terms, but also how we write from different locations. While we hope that other scholars may find some inspiration in our varied experiences, situated in different disciplines and field locations, we would also like to offer students and early career researchers insights on how to concretely manage requirements of transparency in a neoliberal world, as they may experience academic pressure to disclose (Gani and Khan, 2024, p. 1; Reddy and Amer, 2023). In the remainder of this essay, we ask: how do researchers deal with the vulnerabilities resulting from processes of disclosure/exposure? And in particular, how do they engage questions of disclosure and exposure in researching race and racism? In the forthcoming examples, drawn from our conversation during the workshop, reflections on past research, and publications of the coauthors, we disentangle the ways we disclose our identities and the forms of exposure and vulnerability that emerge.

## 2 Theoretical background

To describe the role of disclosure/exposure within academic research, it is important to first situate research, knowledge production, and the university in a broader context. The university and its logics reflect larger societal normativities. Following decolonial authors, who have highlighted the historical roots of power relations in the production of knowledge (e.g., Collyer et al., 2019; Mignolo and Walsh, 2018; Ndlovu-Gatsheni, 2018; Quijano, 2000), we understand the university as a byproduct of modernity, with roots in colonialism and slavery, an era wherein white women could only be subjects, and Black people of any gender were considered objects (Warren, 2018; Hartman, 2016). The driving logic of this system was “predicated on the belief in white European cultural and intellectual superiority over the rest of the world” (Bass, 2025, p. 578). The university is both colonial and characterized by antiblackness, both of which also normalize cisheteropatriarchy (Chipato and Chandler, 2022; paperson, 2017). Processes of othering and ‘stranger-making’ are thus central to the (re)production of higher institutions as ‘white spaces’ (Ahmed, 2012, p. 42) and to the maintenance of gendered power structures, within which non-white bodies, particularly those of women, become hypervisible. This means that certain people (straight white men) are more often considered authorities, and that certain types of research (that which maintains this hegemon) are more legitimate or valid.

These hierarchies also extend to disciplines and disciplinary practice, which prioritize social sciences over fields like Black or Critical Indigenous Studies, and consider theories and methodologies produced in the West as more legitimate or meaningful (Deridder et al., 2022; Myers, 2023; Macharia, 2016; Murphy and Zhu, 2012). Postcolonial power structures influence the entire research process, from research design to implementation, data analysis and publication, and in turn produce *silencing*, defined by Abdelnour and Abu Moghli (2021, p. 3) as ‘the exclusion of marginalized or critical voices, especially those most impacted by violence,’ such as native researchers. Yet, as Harney and Moten (2013) argue, the modern university is itself historically structured through the exclusion of racialized subjects, such that their presence within it often involves a “fugitive” relation—neither fully belonging to nor entirely outside the institution—while the forms of knowledge these scholars produce, though frequently delegitimized, are precisely those upon which critical thought depends It should therefore come as no surprise that the voices that are ‘silenced’ are also the ones being asked to ‘disclose’ more about the conditions of their research and their positionality.

Disclosing one’s identity and positionality means navigating through the meanings of identities both within the communities with which we conduct research, and within academic venues (publications, presentations, within institutions). Self-reflexivity, as Pillow (2010, p. 179) argues, ‘acknowledges the researcher’s role(s) in the construction of the research problem, the research setting and research findings, and highlights the importance of the researcher becoming consciously aware of these factors and thinking through the implications of these factors for her/his research.’ Consequently, the researcher’s race and gender, and those of their research participants, influence how they interact and understand each other, as well as how the resulting data is read by others.

We focus on two aspects of identity in this paper, race and gender, both of which are indicative of the complexities of antiblackness and coloniality on a larger level. In relation to the role of antiblackness, coloniality, and the resulting cisheteropatriarchy, race and gender call for particular attention. Both race and gender are socially constructed: they have shifting meanings over time and space. The embodied and intersectional nature of race and gender (see also Mirza, 2013; Lukate, 2025) partially forecloses the researcher’s ability to disclose, as their physical appearance may reveal identity characteristics (whether or not they are accurately interpreted). Though antiblackness is global, and skin color, hair texture and styles, and other physical features are associated with racial and gendered categories in certain contexts, they are not static constructions. The racial repositioning of Afro-descendant scholars as mixed-race or white in African contexts involves an unsettling process of decentering, where privilege intersects with social discomfort, forcing scholars and interlocutors to position themselves racially and socially differently than they had anticipated (Pereira, 2020; Lukate, 2023). The exposure of physicality results in different forms of disclosure, as researchers may choose to correct assumptions or articulate an identity that was not visibly identifiable.

Exposure, in the context of research on race and gender and with marginalized communities, takes multiple meanings for researchers. To have one’s identity exposed, or to intentionally expose elements of one’s identity, compels researchers to reflect on how their identity is understood both in local and global contexts. In turn, moments of *deliberate* disclosure shape relationships in the field, influence access and trust, and affect how knowledge is produced and interpreted (Harding, 1986; England, 1994; Smith, 1990). In many contexts, researchers can neither hide features of their identity, which seem to indicate a certain gender or race/ethnicity, nor control the way their interlocutors read these features. They become semiotic signs that acquire social meaning with metapragmatic evaluation in a specific sociocultural context (Agha, 2007). Disclosing an intersectionally racialised and gendered identity in writing can influence how research is read and perceived.

Exposure creates vulnerability by exposing the researcher to specific risks. As Sampson et al. (2008) argue, feminist research methods, by emphasizing a focus on reflexivity, research relationships and responsibilities, expose scholars—and particularly female scholars—to greater emotional harm, including risks such as ‘gender and emotional labor in the field; sexual harassment and emotional risk; emotional demands by participants; controlling emotions; leaving the field; consequences of research on personal life; being silenced; […] identifying/discussing gender-related risks in institutions; gender and performance expectations’ (ibid., p. 922). Their work clearly highlights the costs of adopting a reflexive approach in the field, as researchers develop a deeper sense of commitment to (and compassion for) their participants. At the same time, Pillow (2010, p. 273) points out, doing ‘self-reflexivity is most identified with self-disclosure.’ As reflexivity becomes co-constructed through social interactions, it may involve exposing and discussing elements of one’s identity (e.g., gender, race, but also sexual orientation, romantic partnerships, choices regarding dating and family planning) and thus require additional emotional labor. In writing, Pillow (2010) argues, engaging productively with reflexivity involves going beyond the use of reflexivity as a validation or legitimation statement. Yet, again, this comes with specific costs associated with the exposure of personal details to colleagues and readers.

Vulnerability, Gilson (2024, p. 267) states, represents ‘a foundational openness to being affected and altered’—one that is profoundly relational, because it emerges from intersubjectivity. Emphasizing the ethical value of vulnerability to deconstruct the neoliberal paradigm of invulnerability and its associated moral values—namely, power, domination, autonomy and self-reliance among other features—Boublil (2024) argues that vulnerability ‘presupposes [our] openness and exposure to the world and others’ (Boublil, 2018, p. 184). Its embeddedness in ‘intersubjective relations based on trust, care, and responsibility’ (Boublil, 2024, p. 280) makes vulnerability a central element of the ethnographic method. The possibility of being affected thus needs to be collectively acknowledged as an epistemic posture. Nevertheless, even as we consider ‘self-exposure as openness to alterity’ (ibid., p. 278), we also need to consider researchers’ efforts to both establish boundaries and limit exposure.

In our teaching and mentoring practices, we have observed that students who are themselves marginalized often feel greater pressure to disclose: Shall I detail my family story to explain my relation to this immigrant group? Shall I explain that I was myself a porn actor/ress or a sex worker to explain why I chose this topic? Do I really have to write that I am transgender and explain how my transition affected my field research if I do not want to? By contrast, other students feel ready to use reflexivity and personal experience as data but do not know how: To what extent is it possible to use autoethnography? How confessional can I be? (also see Pillow, 2010) While we may offer a nuanced answer to each student, we would like them to know that experienced researchers encounter similar dilemmas regarding disclosure and exposure. Researchers can feel discomfort in disclosing personal information, whether in positionality statements or in front of interlocutors. We can feel vulnerable because we are required to write about ourselves in journal articles, especially at a time when open access policies are becoming widespread and we are faced with the possibility of our words and arguments being available on the world wide web in perpetuity.

### 2.1 Positionality

Our discussion of disclosure, exposure, and positionality evolves from this context to focus on two elements: how we understand positionality, and how we frame positionality in writing. Positionality statements are understood as a reflective strategy across the social sciences. However, and perhaps unsurprisingly in the context of academic research described above, the reflexive process often results from unequal power relations (Pillow, 2003, p. 185), as researchers approach field research from a position of social, racial or gender privilege. Producing reflexive texts allows researchers to express their thought processes and ‘shifting subjectivities’ throughout the research (ibid., p. 189). Many authors have productively interrogated the insider/outsider binary, highlighting instead a continuum of degrees of ‘insiderness’ and ‘outsiderness’ depending on situations, interlocutors, and knowledge of the research context (see, e.g., Bukamal, 2022, Chaudhry, 1997, Narayan, 1993, Quashie, 2017, Yip, 2024). Chaudhry’s text “Researching ‘my people,’ researching myself” (1997) offers a comprehensive example of how, in certain situations, the researcher can experience confusion, contradictions and conflicting allegiances due to the coexistence of divergent (cultural) modes of thought *within them*.

Nevertheless, positionality as a method, as Gani and Khan (2024), point out, has its roots in the dominant ‘coloniality of power’ (Quijano, 2000) that it seeks to counter. As a reflexive methodology, it was ‘founded *for the white researcher* to better conduct his/her research *on the non-white other*, albeit in a more ethical way’ (Gani and Khan, 2024, p. 5). The non-white ‘other’ is the object of study and placed in a subordinate racial position. Non-white researchers (researching non-white communities and individuals) are presented with a choice that presupposes their lack of objectivity: either they situate themselves as social/racial ‘insiders’ to a specific group and must justify how they recognized and avoided ‘bias’ or they explain (aspects of) their ‘outsiderness’ but still run the risk of being criticized for not being ‘neutral’ enough—a conundrum with no satisfactory solution. Indeed, as we shall see, ‘insider’ positions are subject to even more rigorous scrutiny from peers.

A critique emerged in the 1990s against reflexivity and positionality statements as a self-centered, self-indulgent approach to research (see Pillow, 2003, pp. 176–77). While Patai (1994), denounces an excessive concern with representation in women’s studies, feminist cultural theorist Lisa Adkins argues that the practice of reflexivity has been dominated by self-technologies that make the researcher visible in their texts (fieldnotes, confessional style, personal narratives), thus undermining participants’ voices (Adkins, 2002 cited by Swan, 2008: 394–95). In anthropology, Geschiere (2010) warns against the danger of researchers becoming their own subject of writing; as texts are perceived as a literary exercise, the researcher’s ‘imagination and experiences’ (2010, p. 138) tend to take precedence over contextual and material analysis. Self-reflexivity may open up the way to a ‘monologue genre’ that is detrimental to research quality. However, for Adkins (2002), the central issue is that the ability to be reflexive implies mobility in terms of identification, as research is a boundary-crossing process. Identity mobility is thought to be the privilege of some identities (male, white) and not others. She argues that ‘while some social scientists are deemed mobile (and their research hence reflexive and sound), others are deemed fixed (their research unreflexive and hence questionable)’ (Adkins, 2002, p. 346).

Western-centric academic standards of ‘outsiderness’ that attest to the value of research are further reinforced by criticisms against ‘me-search’—a term with pejorative connotations that qualifies studies that are personally relevant to the researcher. In the US context, in particular, the concept of ‘me-search’ has been used to discredit Black sociologists by assuming that personal proximity to the field implies unreliability in data collection and analysis (Harris, 2021). This injunction to ‘neutrality’ extends to the peer-review process, as ‘shared identities with the communities under study are met with accusations of subjective bias, and questions about comparisons to similar white people to “ensure” generalizability’ (ibid., p. 83). These types of accusations concern Black scholars, but also female, LGBTQIA+, Indigenous, and scholars from other minoritized groups, who conduct research on topics linked to their identity, despite a substantial body of literature that highlights ‘the methodological power of shared identities’ (Harris, 2021; see also Collins, 2000). All knowledge is situated, and all researchers study topics of personal interest, to some extent (Haraway, 1988). We therefore reformulate this criticism as actually being positive, because their positionality affords them a unique perspective and personal knowledge of the subject.

### 2.2 Written positionality statements

Written positionality statements, in turn, come with their own set of concerns. Specifically, positionality statements have become an academic trope that mask the complex and dynamic nature of power relations in the field, and also reproduce these relations under the guise of self-accountability (Savolainen et al., 2023, p. 1331). Following Sara Ahmed’s (2012) critique of institutional language, statements of positionality, in their most concise versions, often appear as ‘non-performative’ in the sense that they do not disclose the power relations they seek to analyze. They are stylistic exercises that reveal less about a researcher’s identity in the field *than about their position within academia*. By demonstrating the author’s awareness of critical asymmetries in the research process, positionality statements affirm ‘the epistemological authority assigned uniquely to Western university as the privileged site of knowledge production’ (Bhambra et al., 2018, p. 3). As Harney and Moten (2013) argue, the figure of the ‘critical academic’ does not stand outside the institution they interrogate but is positioned within a university structure that accommodates and repurposes critique. As a consequence, reflexive self-criticism risks reinforcing, rather than disrupting, the very hierarchies it seeks to challenge.

From this perspective, statements of positionality create an ‘illusion of a commitment’ (Ahmed, 2012, p. 130) to greater academic equality and inclusion, without creating the conditions necessary to foster change. They may be read as part of other ‘settler moves to innocence’, which Tuck and Yang (2012, p. 10) define as ‘strategies or positionings that attempt to relieve the settler of feelings of guilt or responsibility without giving up land or power or privilege.’ Narrow positionality statements which list the researcher’s characteristics and/or focus on self-narration, signaling the researcher’s ‘awareness’, tend to reproduce complicity with structures of power instead of producing accountability and responsibility (Skeggs, 2002; Subramani, 2026).

These critiques of academic language highlight the ways in which power structures are embedded in the politics of writing. What is the significance of positionality statements, then, when researchers are racialized and/or gendered ‘others’? What is the academic value of disclosure in the current academic context? What risks does disclosure entail? Following feminist standpoint theory, many authors have addressed the situatedness of their racial and/or gendered identities in the field and produced deep analyses of the constraints and possibilities linked to their subject position (see, e.g., Golde, 1986; Gunaratnam, 2003; Muwwakkil, 2023; Ryan-Flood and Gill, 2010), including researchers questioning the opportunities and limitations of their ‘native’ or ‘insider’ identity (see, e.g., Abu-Lughod, 2008; Beoku-Betts, 1994; Chavez, 2008; Jacobs-Huey, 2002; Narayan, 1993). This rich body of literature offers a critical view of the production of knowledge as ‘inevitably partial, incomplete, and situated’ (Deridder et al., 2022).

However, we cannot help but notice that the imperative of reflexivity weighs more heavily on scholars who do not fit the cisheterosexual white male norm. As individual researchers, we have experienced that the requests for (more detailed) positionality statements tend to be directed more at women, and in particular Black and Indigenous women researchers, who are asked to explain how they avoided ‘bias’ in their research with women, Black and indigenous communities. Positionality statements often appear as statements about differences (and how the white researcher acknowledged and attempted to bridge those differences), which marginalizes the distinct experiences and situatedness of non-white researchers. With regards to positionality statements, the expectation is often for scholars with marginalized identities to disclose more of their personal stories, in order to prove that their work is legitimate, counteracting the assumption that the research and findings are not “objective” and may be biased. Thus, researchers can feel that disclosing the details of their personal story is necessary to ‘justify’ research. Therefore, we wish to question what seems to have become an academic expectation of transparency, but also analyze the risks associated with exposing the multiple dimensions of one’s identity, particularly at the age of AI and open access publishing. Many journals now require positionality statements in methodological sections, and reviewers often question the length and details of these statements. Reviewers may request additional detail or challenge the perceived adequacy of what is disclosed, raising the question: how much does one need to reveal about oneself to be taken seriously? This question is particularly pertinent in discussions on race and racism, where positionality is often scrutinized more heavily and researchers’ may be expected to disclose their own ethnic-racial identity in mainstream terms (e.g., Black, white), thus fixing identifications that might otherwise be fluid, even if such identity labels are less commonly used in the socio-political context in which the research has been conducted. Furthermore, space constraints in journal articles (typically 7,000–9,000 words) limit the depth with which researchers can explore their positionality. The brevity required often leads to reductive statements that flatten the complexities of identity, lived experiences, and how these intersect with the research process. For instance, it may take less words to state that ‘the first author identifies as a Black woman’. However, a more nuanced positionality statement might require and would give space to discuss the role of colorism, texturism, class, language, religion, and nationality in how ‘being Black’ was perceived and negotiated in interactions throughout the research process. Quashie (2017), for instance, describes how, during her research in Senegal, she had to navigate the different ethnic, racial and social identities that her various interlocutors assigned to her: ‘African’, ‘Senegalese’ or ‘local’ (as she spoke Wolof), ‘Westernized mixed-race’ (‘métisse’), ‘bi-cultural’ or ‘Arab’ as processes of racialization are ‘associated with a supposed social classification’ (2017, p. 232). Data analysis, she argues, ‘must account for the multiple identities that emerge in situations of dialogue’ (ibid., p. 253). Yet such depth may not be feasible in a manuscript’s methodology section. On the contrary, researchers may find it difficult to engage with reflexivity beyond a descriptive statement that lists their characteristics, and how they correspond to or differ from those of their participants—or a ‘shopping list’ statement as Folkes (2023, p. 1304) names it. This is one reason why scholars find positionality statements uncomfortable and unsatisfying, as they reduce a dynamic and evolving relationship to a few static sentences.

Written positionality statements and the process of disclosure also change in individual and co-authored papers. In co-authored papers, positionality unfolds in relation to the broader academic context/ meanings and across the identities of the authors. Positionalities are disclosed as elements of a collective, leading to certain forms of disclosure that would otherwise not take place, such as clarifying different ethnicities in a racially homogenous group. In the subsequent discussion we outline the perils of disclosure/ exposure through four sets of examples: names that disclose, the ‘you knows’ and managing projection, disclosing race and gender, and written statements. In each category we provide details on the complexities of race and gender in disclosure processes, and how the diverse meanings of these identities must be navigated during academic research.

## 3 Results

### 3.1 Names that disclose

A few of us observed that first names and surnames could speak for themselves even before questions about identity are asked, with interlocutors quickly inferring their racial/ethnic positioning. For instance, Lukate noticed that some of her interlocutors could tell that her surname indicated family ties to the Democratic Republic of Congo, though her German first name(s) and absence of a second ethnic name often meant that people regarded her as being born outside of the DRC. The congruence of the semiotic signs with other signs, such as hair texture, further identified her as a German woman with both German and Congolese heritage. Her names exposed parts of her identity that she did not have an opportunity to articulate.

Okigbo reported a similar incident during an online interview for a research project on Nigerian immigrants’ marriage decisions (Okigbo, 2023). Okigbo is herself a Nigerian immigrant, and therefore has an intimate understanding of cultural dynamics alongside her academic background. In her research and within Nigerian communities, ethnic identity, which is discernible by name, is associated with other stereotypes. In this case, she had logged into an online platform, without considering that her full name would be visible to her correspondent. She had chosen to keep her ethnic background concealed prior to this interaction, to prevent creating social distance with her interlocutor, but the person recognized her name and identified her ethnic origins at the start of the interview. From Okigbo’s perspective, this incident undermined the neutrality required to collect specific data.

The racialized nature of names may influence researchers at other phases of the research. While the first two authors grappled with the impacts of disclosure after the first exposure of their names, Bass found that her surname obfuscated her racial identity, or implied whiteness. The name itself is the result of her African diasporic identity, albeit one that transited and received its name through a slave plantation in the USA (Hartman, 2016; McKittrick, 2006). In interactions during her research with members of Black and African diaspora organizations in Germany, participants expressed surprise that her racial identity did not match their presumed racial categorization of her name.

The Black community in Germany is composed of Afrodeutsche or Black German people and Black and African migrants (Aikins et al., 2021). In general, Black people in Germany do not have the same connections to the transatlantic slave trade as Black communities in other countries, such as the USA (Wright, 2004). As such, names with European origins emerging from histories of displacement through enslavement are not typically connected to Black people in Germany. While Bass disclosed her actual heritage in response, the exchange demonstrates how her name appeared to disclose a (white European) identity that she corrected by disclosing her personal background. These examples are indicative of the difference between exposure and disclosure, and how researchers must navigate those aspects of their identities and positionalities that are revealed about them through their names. Names both expose us to stereotypes and can disrupt stereotypes, they modify research exchanges, and add another dimension to the disclosure of racialised identities.

### 3.2 The ‘you know’s’ and managing projections

Imagination, representation, and emotions relate to the next point: how to manage interlocutors’ projections of researchers during the research process, or managing what we call the ‘*you know’s*’. While interlocutors underestimate the ability of the researcher to empathize, they can also overestimate their ability to ‘understand’ a specific experience including the ability to navigate specific institutions and policies that are part of their own societies such as immigration laws and specific legal requirements. Interlocutors have ideas about who we are and what we know, and, by inferring our positionality, they also may consider us as the ‘representatives’ of our own society. We can encounter situations where interlocutors project a certain type of knowledge into the researcher and assume that we know, though we do not. For instance, interlocutors may assume that the researcher knows about asylum or other administrative procedures. Managing the ‘*you know’s*’ is about the timely disclosure of the lack of cultural/social skills—and it is not straightforward. It has to do with how comfortable the researcher feels about divulging a lack of skills or knowledge. For example, disclosing a lack of competence in the field places the interlocutors into the role of expert which may lead to them sharing more information with you. By contrast, revealing the same lack in positionality statements can leave the researcher open for criticism.

There are moments during interviews that challenge our position as ‘knowing’ researchers. Ménard, for example, describes how the ‘*you know’s*’ appeared within her research with Sierra Leonean migrant women (Ménard, 2025), as first referenced during our workshop conversation:

During my work on intimacy and relationships in migration, women I worked with considered me as a ‘cultural insider’, as I have lived for a long time in Sierra Leone and I have learned the language (again there is some of my family story involved here). Therefore, they said statements such as ‘you know how it is there’ or ‘you know how African men are’. But I have never been married to a Sierra Leonean and I have only a limited knowledge of marriage patterns in Sierra Leone. Interlocutors project a certain type of knowledge and assume that ‘you know’. It has to do with assuming a certain identity in the field.

As Lukate pointed out during the workshop, there are two options: 1. Capitalize on the ‘outsider’ position and say right away ‘I do not know’ (which is not always possible during interviews or conversations, as it can jeopardize credibility) 2. Acknowledge to yourself that you do not know and ask for clarification later (which the ethnographic method allows to a certain extent). In Ménard’s example above, the researcher chose not to intervene due to the particular emotional context of interviews—which concerned marital relations and violence—but clarified these statements later with the help of other Sierra Leonean women of her participant network. Intervening, in this case, would have created a rupture in self-narration on issues involving self-disclosure, emotional vulnerability, and requiring courage to be expressed.

Lukate took her own research as an example of how to manage disclosure in interactions where the research participant assumes that the researcher has particular knowledge (due to their ethnic background), in response to which the researcher must manage their disclosure:

Interviewee: This is maniok. You know what maniok is?

JML: The root, right? The food?

Interviewee: No. Yes, yes. From your father, yes, maniok is a root but maniok is also a fertilizer.

In this exchange, the interviewee begins with the assumption that Lukate knows what maniok is because of her connection to the African continent. Lukate responds to this assumption passively rather than correcting it, choosing not to further disclose or expose herself. Similarly to Ménard, her silence here also reflects a careful weighing of the benefits and costs of disclosure in an interview situation. Correcting or expanding on her own experiences and knowledge would shift attention away from the interviewee’s story toward the researchers’ biography and may disrupt the narrative flow of the interview. Lukate thus manages the presumed knowledge by letting the participant lead the conversation, giving them space for further inquiry, but also allowing them to simply carry on with their own narrative.

### 3.3 Disclosing race and gender

Questions of what and how to disclose one’s identities are enmeshed in intersectional dynamics and situated in the various gender/racial/social displacements we experience in the field. Before entering any fieldsite, researchers benefit from mapping their own identity positions and anticipating which identities may become salient, and how. During fieldwork, researchers benefit from keeping a journal and reflexively revisiting this identity map to track which identities became more prominent, and when. Drawing on our own diverse research experiences, we share four broad disclosure strategies researchers may draw on:

Proactive disclosure: volunteering identity information to establish trust or legitimacy.

Responsive disclosure: sharing information about one’s identity, heritage and personal experiences only when directly asked by participants.

Strategic omission: allowing a salient identity (e.g., race, nationality) to foreground itself while leaving others unaddressed.

Deflection: redirecting personal questions without explicit disclosure or denial.

No strategy is value neutral and researchers may find that they engage in all strategies during the research process. Crucially, each strategy shapes relationships in the fieldwork context in different ways. The three vignettes below illustrate how these choices unfold under real field conditions, and offer guidance for researchers struggling to navigate risks of exposure and vulnerability.

*Vignette 1: Gendered disclosure and the question of motherhood*: In working mostly with women, Ménard, Scarabello and Lukate each noticed in their respective fieldwork that participants used questions about motherhood and romantic partnerships to establish connections and trust. Women wanted to know whether the researchers had children, and if so, how old or what gender, and whether their partner was from the same cultural/ethnic background. These questions were not borne out of pure curiosity, but a way of assessing whether certain topics could be discussed without fear of being judged. Critically, the racial identity of our children was relevant: raising racialized children created different forms of alliances, reciprocity, and intimacy during fieldwork, and enabled the recognition of the expertise of our interlocutors in navigating, resisting and challenging racial hierarchies. Establishing woman-to-woman intimacy then became a way of managing access, even though the exchange of personal experiences on motherhood and romantic relationships operated alongside other power differentials including race and class. Interestingly, while motherhood continues to be stigmatized in academia, or used to systematically disenfranchise women from career advancement, the three researchers acknowledged that their experiences as mothers enabled them to establish connections with women in other contexts. Their status as mothers was recognized and appreciated by research participants, who saw this as an opportunity for deeper and shared understanding.

*What this means for researchers:* Gendered expectations and personal disclosure are central to negotiating access and belonging in the field, yet opening up may interfere in fieldwork relationships precisely when those gendered and racialized expectations do not align with one’s personal circumstances and life choices. Participants often use personal questions as tools to establish whether they can trust the researcher: *what do I (as the participant) feel comfortable sharing with this researcher? Can they empathize with my position / experience / decision, and if not, do I want to continue this conversation?* You will be asked questions about aspects of your life (e.g., family, religion, relationships, education) that you may not have anticipated disclosing or discussing. Before fieldwork, identify which personal identities or experiences you are willing to share, and, importantly, which you are not, and what you will say when asked about those you prefer not to disclose. Silence and deflection are themselves communicative acts that will be noticed and interpreted by interlocutors. Proactive disclosure of some identities and personal information (e.g., marital status, number of children) can facilitate relationship building and open doors. However, it can also invite further questions (e.g., child phenotype, miscarriages) that you do not feel comfortable.

*Vignette 2: Shared history and language as partial bridge:* In conducting research in Cape Verde and Guinea-Bissau, Venancio noticed how being Brazilian was important to establishing connections and gaining access to the field. In this context, Brazil, Cape Verde and Guinea-Bissau were seen as “sibling-nations” due to Portuguese colonialism, which allowed for the researcher and research participants to interact from a shared sense of kinship and familiarity. At the same time, not being born in an African country put him in a clear outsider position that offered an unusual degree of social latitude, including access to female domestic spaces and interactions not typically available to local men. This, in turn, meant that questions about the researcher’s identity and personal relationships were interpreted through the lens of foreign difference rather than social norms. This experience reveals how shared linguistic and historical connections can simultaneously foster identification while also feeding into boundaries of difference and exclusion.

*What this means for researchers*: Shared cultural, linguistic or historical connections can be a genuine asset in the field. However, they rarely map neatly onto insider/outsider boundaries (see, e.g., Beoku-Betts, 1994; Bukamal, 2022; Narayan, 1993; Yip, 2024). Prior to entering the field, consider which aspects of a shared identity you will foreground, whilst remaining attentive to how participants welcome and qualify that connection. Choosing an active disclosure strategy may enable the researcher to establish legitimacy with research participants. Proactive disclosure of heritage thus can build rapport but also create expectations of solidarity that complicate your role as a researcher.

*Vignette 3: Gender and the politics of asking questions about the researcher:* In researching identity transformation in the context of migration from the African continent to Germany, Lukate adopted a responsive approach to disclosure, introducing herself as a researcher interested in learning about the interviewees’ migration experience. This approach produced a striking, gendered asymmetry: female research participants frequently and openly asked questions about Lukate’s background, family, and motivations, while male interviewees rarely asked personal questions, usually signing the consent form and moving directly onto the interview. This pattern echoes Clerge (2019) observations, where she found that women were not only more likely to participate in an interview but were also more willing to meet alone, while men often asked for their wives to be present during the interviews, thus managing the social optics of meeting with a young female researcher in private. In both Lukate’s and Clergé’s fieldwork, gender thus structured not just what participants were willing to share, but whether and how they engaged with the researcher as a person.

This asymmetry is not accidental. Instead it reflects the process of “doing gender” in research (West and Zimmerman, 1987). For women, relational talk is a culturally conditioned mode of building trust and connections. Thus, female participants may ask more, and more personal questions because they are accustomed to reciprocal disclosure in intimate conversations, but also because they want to assess whether they can trust the researcher with sensitive topics. Male interviewee’s reservation or hesitation, in turn, may reflect both a greater comfort with transactional interactions and, as Clergé notes, a heightened awareness of the social surveillance and scrutiny that may follow cross-gender interview situations. Importantly, Lukate noticed that, despite or in spite of not knowing the researcher very well, men were often more forthcoming during the interview and shared more narrative information.

*What this means for researchers*: Research encounters are structured by the very social categories we study. Depending on who asks, what they ask, and what assumptions they make, your identity will be differently constructed and perceived. A responsive or passive disclosure strategy can give participants more control over the relational dynamic and shift the focus of the interview from the researcher to the participant. However, this approach can produce inconsistencies. It also does not protect you from disclosure, it merely defers it.

Race and gender are dynamic and complex positionalities for researchers to live and think through, and the process of disclosing makes the researcher deal with varying, and at times oppositional, concerns. In each of the above examples, researchers chose a form of disclosure in relation to their individual identity and the focus of their research. Importantly, as researchers, we did not follow fixed scripts but made fluid judgments about the disclosure process. Based on our collective experiences around disclosure and exposure during fieldwork, here are five practical takeaways to help manage identities and positionality throughout the research process:

1. Map your identities before you enter the field. Taking into account both who you are (your body and how you present yourself to the world) and the topic of your research, which of your identities may be salient in or important to your field site? Which aspects of your identity might be unexpected sources of connection, and which could cause friction?
2. Decide in advance but remain open to answer / respond to unexpected and uncomfortable questions. Consider which disclosure strategy you will default to, while accepting that participants’ curiosity and questions may require you to reveal or talk about aspects of yourself or your life that you are hesitant to share.
3. Prepare for the questions you feel uncomfortable answering. Being aware of topics you are uncomfortable discussing can prevent you from being caught off guard. Prepare a short and honest reply for questions you would rather not answer.
4. Debrief and document. Keep fieldwork notes on moments of disclosure/exposure. Take note of what you shared and what you withheld, how the information shared shaped the interaction, and how you felt about the question and your answer. These notes are analytically useful and may facilitate reflexivity during the write up stage.
5. Find a circle for collective reflections and support. Seek out a supervisor, mentor, or peer group with whom you can discuss not just the intellectual challenges of your research but the emotional labor that goes into meeting people in the field and conducting research. If your institution does not offer this formally, build it informally through a writing group or a standing agreement with a supervisor. The integration of reflective supervision and emotional debriefing into research practice can ensure both the ethical integrity of your research and your wellbeing.

### 3.4 Written statements

Written positionality statements offer a different set of possibilities and challenges to exposure and disclosure than verbal or physical expressions. While disclosing identity during interpersonal interactions and ethnographic research has nuance and flow, a written statement is a static documentation that is publicly available. Researchers must navigate a double standard of anonymity: while protecting interlocutors through practices such as anonymization is an obvious ethical priority, researchers’ own identities may become publicly accessible, particularly in the digital age. This places researchers in an exposed position, where personal details–whether voluntarily shared or inferred–become part of the academic and public record. We do not seek to dismiss the importance of positionality in research, but we do question the extent to which researchers are obliged to disclose personal information, particularly when such disclosures produce discomfort or carry (unintended) risks for researchers personal wellbeing, public standing, and their safety.

Ménard, for example, shared that in writing her book on local practices of reciprocity in Sierra Leone (Ménard, 2023), she omitted to mention years of childhood in Sierra Leone, because it touched on personal details of her life that she did not want to disclose. Had she been truly transparent, she should have disclosed that this impacted the way people perceived her presence and their openness toward her. One specific event—her father’s visit to Sierra Leone in 2012—further cemented these relations. However, the formalized publication of these intimate details brought emotional risks that she did not want to be exposed to. She put scattered elements of this story in her acknowledgements, but without linking them explicitly to power relations and reciprocity in the field. Although this omission was not entirely satisfactory, it nevertheless seemed necessary in this context. She also noticed that many anthropologists have personal ties to the country in which they conduct their fieldwork, but rarely choose to mention or explain them.

To demonstrate the challenges of disclosing positionality in written statements, we discuss how researchers differentially manage disclosure and exposure in the field and in writing. The transition from lived encounters to published narratives requires continual negotiation between authenticity, self-protection, and disciplinary conventions. Returning to Vignette 1, one researcher shared that, “in conversations with interviewees” she speaks about her partner and raising her children. While this disclosure feels natural during dialogue with research participants, such personal details evoke feelings of discomfort and unease when included in written texts. During our workshop, we collectively worked on how to write about such personal details, suggesting that she could paraphrase the details (e.g., age of children, personal information about their partner) in a more abstract form, e.g., “*Motherhood and marriage as a shared experience between the interviewees and the researcher shaped conversations, facilitating trust and establishing shared experiences*.” This revision retains reflexivity and acknowledges the researcher’s positionality while reducing exposure and emotional vulnerability. The contrast between these two formulations highlights how researchers can balance transparency with discretion, and use abstraction or generalization to convey the role of their positionalities within the research process without overexposing their private life in written publications.

Similar considerations arise when reflecting on racialized identities. In Lukate (2022), the author writes: “My identity as a self-identifying German mixed-race female academic […]. While I matched interview participants by gender, I had to continuously negotiate my status as a Black German “mixed-race” woman with “good hair” in interview situations and ethnographic settings, particularly where issues of colorism and texturism were raised.” This detailed account illustrates how specific aspects of her racialized and gendered identity shaped access, trust, and the research process itself. Moreover, her attention to details such as nationality and hair type is rooted in an understanding of their influence on the data collection process while researching the psychology of Black hair (Lukate, 2019, 2025). For an academic audience that may be unfamiliar with Blackness and its multitudes, Lukate makes clear in her statement how her positionality functions in the research context. However, in a later co-authored publication, her positionality is framed more broadly: “The second author identifies as a German woman who has lived in Germany, England, and the United States. Her identity, as well as her research interest in the nuances of identity formation and social categorization across different societies, are shaped by her own experience growing up in a bicultural family in Germany and exposure to experiences of racialization in different places around the world.” This approach communicates reflexivity about racialized experience without delving into the role of physical traits, which reduces discomfort and exposure, particularly in European academic contexts where open discussions of race remain sensitive. However, it also reflects the particularities of the articles themselves (an empirical paper discussing interview and fieldwork data versus a co-authored conceptual paper) and the ways in which different aspects of identity mattered while writing each paper.

In a related example, Bass (2022, p. 3) discloses her identity beyond a simple racial categorization to demonstrate to the academic audience how her racialized positionality functions in research. In an ethnographic description of an Oromo women’s protest, Bass clarifies that “while this analysis draws on historical and scientific sources, it is also colored by my own experience as a Black woman who has walked the streets of Oromia and Germany in turn, and who knows intimately what the disdain of the empire feels like”. In this example, Bass (2022, p. 3) situates her raced and gendered identity (Black woman) alongside her relationalities and geographies (“the streets of Oromia and Germany”). In other parts of the paper, she also describes herself as a “friend and comrade” who attended the march as an act of “lived solidarity” (Bass, 2022, pp. 2–3). These details give clarification about the role of her gendered positionality, as a woman attending a women’s protest. Through disclosing these relationalities, Bass makes clear that her shared racial categorization is also impacted by other factors.

Across these examples, we see that positionality statements often shift over time: from intimate field disclosure to more abstracted, context-sensitive narratives in written texts. Based on our reflections, we highlight four strategies for managing written positionality statements: (1) abstract personal details while retaining analytical relevance; (2) situate relationally, connecting your identity to participants, context, and power dynamics; (3) adjust disclosure over time: field interactions, workshop discussion, and written outputs each require different levels of personal detail and revelation; (4) recognize that positionality is multidimensional and intersectional. Taken together, these strategies help manage both ethical concerns and personal discomfort.

By comparison, in co-authored papers decisions become collective; authors must negotiate multiple positionalities and comfort levels, balancing individual reflexivity with shared responsibilities to present the research context. This adds an additional layer to the micro-politics of disclosure, requiring care and coordination. Co-written and co-authored papers force the researcher to describe their positionality in relation to academic and societal definitions of their identities, and to the influence of these norms on the intimate interactions between research partners.

For instance, in revisiting positionality statements that we have written in the past, some of us noticed that in a paper on racialization, anti-Blackness, and whiteness (Moffitt et al., 2025), the EU-based authors chose not to explicitly name their racial identities, instead describing bicultural backgrounds and experiences tied into particular national contexts. Notably, only the white US-based author identified her racial identity, highlighting how naming, or withholding, racialized positionalities can reflect active choice against the inherent antiblackness of academia and academic publishing, which usually forces marginalized and racialized scholars to disclose, and allows whiteness as the invisible norm.

The second example of co-authorship comes from a paper written by a homogenous racial and gendered group of authors. Their general shared positionality is described early in the paper as a group of Black women. However, in the paper’s Positionalities section, this homogeneity is explicated via the sociopolitical context and experiences of Black women in Europe more broadly, before their individual ways of being Black are described. This form of disclosure exposes privileges and vulnerabilities that would be lost in a more generic or broad positionality statement.

We write as Black people in Germany, but not Black Germans. We write as African diasporic peoples whose connections to Africa are both linked to very specific geographies (e.g., born in the Ivory Coast to Chadian parents) and paths of im/migration and movement that follow in the wake of slave routes. It is also critical to include that our physical movements across Germany and Europe at large are characterized by our physicality as young women living without disabilities,’ even though we access juridical frameworks and infrastructural environments built for white, cisgender men. (Bass et al., 2023, p. 61)

The authors understand Blackness as globally influenced by nationality and migration, and disclose elements of their specific Black geographic positions as a way to expose this. Their racial identities as Black diasporic people give them a different perspective than their Black German friends or co-conspirators. Disclosure here functions to contest the academic and sociopolitical tendency to homogenize Black experiences in Europe, by instead affirming Black German identity. Through the terms of their disclosure, the authors also situate their positionalities alongside contrasting vulnerabilities. Connecting Blackness and African diasporic identities both to individuals born on the African continent (“in the Ivory Coast to Chadian parents”) and to those whose identities unfolded “in the wake of slave routes” is an acknowledgement of the histories of antiblackness. By describing their positionalities and racial identities as developed in this historical context, the authors make clear the homogeneity of Blackness within their group perspective.

This paper differs from the previous examples (Lukate, 2022, 2019; Moffitt et al., 2025) in its disciplinary grounding and in what positionality is understood to *do* in the context of the research and in writing. The first two examples were written by psychologists working within critical and decolonial frameworks, for whom positionality is not a procedural formality or a means of justifying scientific practice, but an epistemological commitment and a way of locating knowlegde production within structures of power, and of being accountable to the communities whose lives and identities are at the center of their research. In the third example, which uses Black Studies as its theoretical base, that epistemological orientation is pushed further and the terms in which identities are understood reflect the disciplinary lexicon of Blackness. Together, the three examples demonstrate how disclosing race and gender in writing is shaped by disciplinary commitments and conventions and interacts with the larger meanings of these identities for individuals, co-authors, and academic audiences.

## 4 The collective as protection: what we learned from writing together

In thinking with and through our own discomfort, we have discussed a number of examples of how researchers can navigate the complexity of disclosure/exposure in the field and in writing. The complicated meanings of race and gender not only shape our research questions and provide points of convergence but underwrite the very processes of disclosure and exposure we describe here, encouraging researchers to make careful and critical decisions.

Bringing together our various perspectives, we would like to emphasize the highly ambivalent nature of disclosure practices in researching and writing, whereby voluntary sharing can become a form of exposure and result in feelings of vulnerability. In the field, exposure appears as a structural element of relationships: personal features (e.g., the color of our skin, gendered aspects of our body, age etc.) are interpreted within existing systems of power and differentiation and in ways that we may have little to no control over. Moreover, while choosing disclosure can represent an openness to more ‘genuine’ human interactions and help build rapport, it can also place the researcher in situations where personal information is disclosed and/or commented on unwillingly. Following Skeggs (2002), writing about interactional dynamics throughout the research process shifts the focus from the reflexive self to larger issues of ethics, accountability and responsibility in research. We strongly advocate that positionality statements reflect this tension between disclosure and exposure as an inherent, processual and interactional feature of fieldwork that reflect *and* shape power relations.

More importantly, we would like to draw attention to how risks and vulnerability associated with exposure are unevenly distributed within research contexts, particularly in writing. Expectations around disclosure in academic writing are shaped by institutional norms, reviewer demands, and broader disciplinary conventions, all of which have implications for researchers’ feelings of vulnerability and perceived legitimacy. Discussing what we had seen, heard, and struggled to write about showed us the intellectual and emotional load that each of us, and often in isolation, had been carrying. Crucially, collective peer discussions, we found, is one of the most productive strategies for researchers to work out what they actually feel comfortable disclosing, and in what form.

This is particularly important in the context of peer reviews. Our discussion highlighted how academic power structures compel marginalized researchers to disclose and expose (in writing) personal information as a means of proving scientific rigor and as a condition for being taken seriously. Individually, most of us had sensed that we were being asked to do something that was not being asked of other researchers in the same way. However, that sense was difficult to name, easy to dismiss as personal oversensitivity, and impossible to verify in isolation. It was only through collective discussion that what we experienced as a private, individual discomfort became recognizable as a shared structural experience: those among us who have intersectionally gendered and racialised identities more frequently faced requests to provide more detailed accounts of positionality, more explicit engagement with their own racialized or gendered experiences, or more candid reflection on how cultural ties to the community that we were researching shaped analytical choices. This observation in itself is a methodological point: the current neoliberal demand for reflexivity and transparency falls unequally on women and ethnically or racially marginalized researchers, especially those using feminist research methodologies (see Sampson et al., 2008), thereby amplifying their exposure to risks including—but not limited to—unwanted scrutiny of the researcher’s personal life, professional delegitimization, and the emotional costs of being read through stereotypes. In this vein, positionality statements become a means of demonstrating the credentials and rigor that is often simply assumed or taken for granted in researchers who occupy more powerful social positions.

Protection (of field participants) and transparency (of researchers) have become part of an institutional language that builds on structural asymmetry instead of advancing solutions for a more equitable academic field (Ahmed, 2012). The demand for transparency is framed as methodologically progressive and ethical. And while such requests often reflect good intentions and genuine methodological concerns, they frequently arrive without an understanding of what such calls for disclosure might cost the author. We thus want to draw attention to what those risks and vulnerabilities actually consist of for researchers occupying marginalized social positions.

Firstly, refusing or limiting disclosure risks being read as evasive or lacking methodological rigor. Early career researchers, in particular, and those under pressure to publish more (faster), may hesitate to push back against reviewers who can reject a manuscript at any stage of the review and publication process. Instead, researchers may feel compelled to address feedback and make explicit certain dimensions of their positionality in order to secure publication.

Second, the costs of disclosure can be real and cannot always be fully anticipated at the time of writing. On one hand, complying with expectations of disclosure may mean revealing more than feels safe or comfortable—about one’s family, identity, political position, or heritage—in a published text that cannot be retracted. Consider, for example, the personal and professional risks (e.g., stigmatization, disavowal by family, loss of friendships) faced by early career researchers who disclose a stigmatized or ‘closeted’ identity; or the risks faced by native researchers conducting ethnography in illiberal contexts (e.g., political persecution, legal and penal threats, fear and anxiety) while studying and working on temporary visas (see Jumet and Mekouar, 2025); or the situation of a researcher who writes about their family background, migration history, or racialised experience in a positionality statement and cannot un-reveal that information at a later point. On the other hand, academic publishing implies a level of temporal permanence in what is being exposed, to audiences far beyond the control of the author. For instance, in the case of political persecution, risks may be anticipatory, as a change in political regime might expose native researchers at a later point. Institutions demanding transparency often do not or cannot offer sufficient shelter and protection including editorial guidance on the risks of exposure and disclosure or permanent positions. Thus, in a neoliberal academic system, the risks of exposure and disclosure are individualized, while norms are collectivized, leaving the structural conditions that make demands for transparency uncomfortable—or even dangerous for some—unaddressed.

We recognize that the critique of positionality statements may lead some authors to refuse or forgo writing them altogether (see Gani and Khan, 2024; Savolainen et al., 2023). However, refusal can constitute a risky strategy, and it is not equally available to all researchers. In this context, having a community of peers, or an experienced mentor, with whom to think through such requests before responding is therefore not merely a question of support but a strategy for survival in the neoliberal academy (Reddy and Amer, 2023). Peer discussion creates a space for what we call *collaborative calibration*: the collective process of working out what is appropriate to disclose, what is necessary, and what is too much. Engaging in such reflections collectively may be easier and provide additional reassurance and greater confidence than doing such reflections alone. *What this means for researchers*: find yourself a peer group or mentor with whom you can share some of the burdens that come with conducting and writing about your research. In such a setting, uncomfortable things can hopefully be said safely and the cost of different choices can be assessed honestly. As we have experienced, both collective discussions about disclosure, exposure and vulnerability in academic fields and multi-voice writing offer a way to mitigate or redistribute the risks of individual exposure.

## Data Availability

The datasets presented in this article are not readily available due to the reflexive nature of the paper. Requests to access the datasets should be directed to the corresponding author.

## Footnotes

1 Two research groups were involved in the organization of the workshop: “Migration, Identity and Blackness in Europe” based at the Max Planck Institute for the Study of Religious and Ethnic Diversity (host institution for the workshop) and headed by Johanna Lukate, and “Gender, Migration and Social Mobility among West African Women in Europe” based at the Max Planck Institute for Social Anthropology and headed by Anaïs Ménard. NOTE: The Max Planck for the Study of Religious and Ethnic Diversity has since been renamed into the Max Planck Institute for Political and Social Science.
